# NOVEL INSIGHTS INTO GAIT, MOTOR CONTROL, AND THE BRAIN: IMPLICATIONS FOR COGNITIVE AGING

**DOI:** 10.1093/geroni/igac059.1308

**Published:** 2022-12-20

**Authors:** Andrea Rosso, Sarah Fraser, Roee Holtzer, Talia Salzman, Caterina Rosano, Emma Baillargeon, Briana Sprague, Qu Tian

**Affiliations:** University of Pittsburgh, Pittsburgh, Pennsylvania, United States; University of Ottawa, Ottawa, Ontario, Canada; Yeshiva University and Albert Einstein College of Medicine, New York, New York, United States; University of Ottawa, Ottawa, Ontario, Canada; University of Pittsburgh, Pittsburgh, Pennsylvania, United States; University of Pittsburgh, Pittsburgh, Pennsylvania, United States; University of Pittsburgh, Pittsburgh, Pennsylvania, United States; National Institutes on Aging, Baltimore, Maryland, United States

## Abstract

What can muscles, gait, and brain activity tell us about cognitive decline? Are there certain markers we can track that are early predictors of cognitive status years later? In the current symposia, our goal is to address these questions with recent pilot, longitudinal and cross-sectional studies that measure potential markers of cognitive decline in different older adult cohorts. The first speaker will present findings on changes in skeletal muscle adiposity and 10-year change in global cognition from the Health Aging and Body Composition (Health ABC) study. The second speaker explores cross-national comparisons of gait speed and its association with cognitive function from the Cohort Studies of Memory in an International Consortium (COSMIC). The third speaker investigates changes in brain activity with functional near infra-red spectroscopy (fNIRS) from single to dual-task walking and its relation to changes in several gait quality parameters. The fourth speaker presents pilot work that examines dual-task gait and tapping with fNIRS and compares the dual-task performance and brain activity of older adults who report experiencing subjective cognitive decline to those that do not. The final speaker presents findings from the Baltimore Longitudinal Study on Aging (BLSA) that demonstrate that early markers of slow gait and metabolic dysfunction could identify those at risk of progression to dementia 7 years prior to onset. Taken together, the findings from this symposium present novel markers of changes in cognitive function in older adults and ultimately targets for prevention or slowing of cognitive declines in older adults at risk for dementia.

